# Recombinant Type III Humanized Collagen Solution for Injection Improves Skin Photoaging in Chinese Population: A Case Series

**DOI:** 10.1111/jocd.70276

**Published:** 2025-07-21

**Authors:** Lin Tao, Yu Yi

**Affiliations:** ^1^ Department of Plastic Surgery Time Plastic Surgery Hospital Shanghai China; ^2^ Department of Plastic Surgery JingRen Clinic Shanghai China

**Keywords:** Chinese population, photoaging, recombinant type III humanized collagen solution, skin rejuvenation

## Abstract

**Background:**

Photoaging leads to structural changes in the skin, such as reduced collagen production, contributing to wrinkles, reduced elasticity, and dyspigmentation. Collagen‐based treatments have shown promise in skin rejuvenation, but the effectiveness of recombinant type III humanized collagen (RhCol‐III) solution for injection in Asian populations has not been widely studied.

**Aims:**

To evaluate the efficacy of RhCol‐III solution injections in improving skin quality for rejuvenation among Chinese cases.

**Methods:**

Eight Chinese participants with Fitzpatrick phototype III/IV, presenting with signs of skin photoaging, underwent once a month intradermal injections of 2 mg/mL RhCol‐III solution over a 90‐day period. Clinical assessments, including VISIA imaging, ultrasound, cutometer, and self‐assessed Global Aesthetic Improvement Scale (GAIS) scores, were conducted at baseline, 30, 60, and 90 days postinjection. Key skin parameters such as elasticity, firmness, wrinkle severity, pigmentation, and pore size were evaluated to assess the effectiveness of the RhCol‐III solution.

**Results:**

Participants demonstrated significant improvements in skin quality, although variable due to individual response. VISIA imaging indicated enhanced skin tone evenness and radiance. Participants who had visible wrinkle reduction and pore size decrease, on further assessment, were found to have increased dermal thickness and density, along with improved skin elasticity and firmness. Most participants reported high levels of satisfaction with the treatment outcomes, and no significant adverse events were observed.

**Conclusions:**

RhCol‐III solution injections appear to be a safe and effective treatment for skin rejuvenation, offering improvements in elasticity, firmness, skin tone, and radiance. This case series brings out the potential of RhCol‐III solution in providing substantial anti‐photoaging benefits in Chinese facial skin.

## 1 Background

Aging is a complex process involving degenerative changes in all skin layers, with the most noticeable changes occurring in the dermis. Dermal atrophy is caused by a reduction in extracellular matrix (ECM), particularly a decrease in collagen, which is the main determinant of skin's antiaging ability. In human skin, types I, III, and V collagen make up 80%–90%, 8%–12%, and 5% of the total collagen, respectively. Type III collagen is essential for maintaining the structural integrity and elasticity of connective tissues. However, aging leads to decreased production and increased breakdown of type III collagen. This reduction results in decreased quantity and quality of collagen, causing tissues to become stiffer and less flexible, which contributes to visible signs of aging such as wrinkles and fine lines [1, 2].

Skin aging is influenced by a combination of intrinsic and extrinsic factors. Intrinsic aging is genetically predetermined and involves the gradual decline of cellular functions, where cells lose their ability to divide and repair themselves over time. This is often linked to telomere shortening, which limits cell replication. Extrinsic aging, on the other hand, results from environmental factors such as sun exposure, pollution, and lifestyle choices, like diet and exercise. Most importantly, premature skin aging caused by ultraviolet (UV) damage from sun exposure is known as photoaging, which results in coarse wrinkles, skin atrophy, skin laxity, rough texture, dryness, telangiectasia, yellowing, skin sensitivity, and dermis density changes [3, 4, 5].

Photoaging in Asian populations exhibits distinct characteristics compared to Caucasian skin. Skin in Asian populations is mainly characterized by an increased epidermal melanin content, conferring relatively higher resistance to photodamage. Thus, classic clinical signs of photoaging, such as wrinkles and rhytides, tend to appear later in life (around age 50), as compared to lighter‐skinned individuals. Furthermore, Asian skin is particularly susceptible to UVA‐induced pigmentation, as demonstrated by a significant correlation between wrinkles and dyspigmentation in both Asian men and women [6, 7, 8, 9].

In addition to photoprotection, several treatment methods are available for managing skin aging. These include topical agents, such as retinoic acid, ascorbic acid, glycolic acid, and peptides, which target various aspects of skin rejuvenation. Energy‐based devices, including lasers, radiofrequency, and ultrasound, are also employed to improve skin texture and tone by stimulating collagen production and enhancing skin elasticity. Additionally, dermal fillers are used to restore volume and reduce the appearance of wrinkles and fine lines [3, 4, 5].

Topical medications, while effective, require continuous use for 3–6 months to achieve maximum effect. Participant adherence may be a challenge due to the delayed onset of visible results and potential skin irritation or dryness. Laser or intense pulsed light (IPL) treatments offer relatively fast results in skin tightening and collagen remodeling. However, they often come with relatively low tolerability, including discomfort during the procedure, and there is a higher risk of postinflammatory hyperpigmentation in Asian populations [9]. Hyaluronic acid fillers may cause side effects such as swelling, bruising, or granuloma formation. Additionally, many currently available collagen fillers are derived from animal sources, which can lead to allergic reactions and other complications [3, 4, 5].

Recombinant humanized type III collagen (RhCol‐III) solution for injection represents an innovative approach to skin rejuvenation and repair. Unlike traditional animal‐origin collagen products, RhCol‐III is produced through recombinant technology, ensuring a higher degree of purity and reducing the risk of allergic reactions or zoonotic transmission [10, 11].

As an antiaging product, RhCol‐III solution offers multiple benefits for skin health, including directly increasing collagen content within the dermis, improving skin elasticity, and enhancing skin tone brightness [12, 13, 14]. In terms of skin repair, RhCol‐III integrates seamlessly into the ECM, supporting tissue remodeling. It stimulates dermal fibroblasts, the primary cells responsible for producing collagen, hyaluronic acid, and other ECM components, thereby promoting neocollagenesis and improving overall skin structure and resilience [15, 16, 17].

Here, we report a case series of eight participants who received RhCol‐III solution treatment and their respective improvements in skin quality.

## 2 Methods

### 2.1 Case Series Participants

A total of eight cases, three males and five females with age ranging from 31 to 44 years old who were seeking aesthetic skin quality improvements and who met the eligibility criteria were analyzed. Cases were eligible for inclusion if they (1) presented with at least one of the following: hyperkinetic lines or wrinkles (forehead, crow's feet, glabellar), poor skin quality characterized by dry lines, fine lines, dark skin tone, photoaging, or enlarged pores, acne scarring in a stable period, and sensitive skin; (2) voluntarily consented to receive intradermal injections of recombinant type III humanized collagen (RhCol‐III) solution after being informed of all associated risks. Cases were excluded if they (1) presented with signs of infection, open wounds, or active acne on the facial skin; (2) underwent laser procedures, chemical peels, or any other facial procedures that could impact the dermal layer within 3 months prior to the study; (3) were recently treated with injectable dermatological or cosmetic agents (such as botulinum toxin or hyaluronic acid) on the face within 6 months of enrollment; (4) in the period of menstruation, pregnancy, or lactation; (5) had a history of allergies to collagen products, RhCol‐III injection ingredients, or local anesthetics; and (6) had a history of autoimmune diseases, use of immunosuppressive medications, coagulative disorders, anticoagulant therapy, or severe heart or kidney dysfunction.

### 2.2 Administration Technique of RhCol‐III Solution Injection

After cleansing the skin with a mild cleanser, topical anesthesia (lidocaine and prilocaine) was applied for 30 min. Following the removal of anesthesia, the skin was disinfected with iodine. At 30, 60, and 90 days after baseline, cases received 8–10 mL of recombinant humanized type III collagen solution for injection (RhCol‐III; NMPA medical device registration number: 20233131245) RhCol‐III solution each time. Upon the evaluation of periorbital skin aging extent by an experienced clinician, a 34‐Gauge, 4‐mm needle was used to deliver 1–2 mL of RhCol‐III solution to each periorbital area. The remaining 6 mL of the solution was injected bilaterally in the zygomatic and forehead areas with an electronic injector set to a dose of 0.025 mL per injection, slow speed, 10% negative pressure, and 80% retraction force. Injections targeted 0.8–1.2 mm depth for the cheeks and 0.6–0.8 mm for the forehead area.

Participants were advised to apply a collagen or hyaluronate‐containing face dressing once or twice daily within 3 days postinjection. Starting 3 days after the procedure, a daily moisturizer was recommended. Additionally, participants were encouraged to maintain a healthy lifestyle, including good sleeping habits, minimal alcohol intake, reduced sun exposure, and moderate exercise.

### 2.3 Outcome Evaluation of RhCol‐III Solution Injection

Skin quality was evaluated using the four parameters defined by a Global Advisory Board—skin tone evenness, skin surface evenness, skin firmness, and skin glow [18]. Overall skin quality was assessed via high‐resolution VISIA (Canfield Scientific) photographs taken at baseline and 30, 60, and 90 days posttreatment. VISIA images were analyzed with IPP Plus (Media Cybernetics Inc.) imaging analysis software to evaluate skin glow (skin radiance and Individual Typology Angle, ITA), skin tone evenness, and skin surface smoothness (wrinkles and pores). Cheek dermis density and thickness were quantified with UC22 (Courage+Khazaka Electronic GmbH), and skin firmness and elasticity were measured with a cutometer (Courage+Khazaka Electronic GmbH). Participant satisfaction was assessed by Global Aesthetic Improvement Scale (GAIS) scores at 90 days after baseline.

### 2.4 Ethical Considerations

The case series was conducted following the principles of the World Medical Association Declaration of Helsinki. Written informed consent for the use of participant photographs in publications of study results was obtained from all participants prior to taking part in the study. Participant privacy rights were protected in accordance with ethical guidelines.

## 3 Results

### 3.1 Overall Case Series Presentation

A series of eight cases were analyzed, with a mean age of 36.9, including three males (37.5%) and five females (62.5%), all of Han ethnicity with Fitzpatrick skin phototypes III/IV, presenting a range of skin concerns such as wrinkles, uneven skin tone, enlarged pores, skin laxity, and hyperpigmentation. Most had oily or mixed skin types, except for one with sensitive skin. Skincare routines varied from simple to complex, with all participants reporting excellent sunscreen adherence. Sun exposure was minimal to moderate. Several participants had a history of prior aesthetic procedures, such as IPL, RF, or laser treatments, with varying timelines, whereas one participant reported no previous aesthetic procedures (Table 1).

**TABLE 1:** Baseline data and clinical evaluation of eight cases treated with RhCol‐III solution injection.

Case	Age	Sex	Skin type	Skincare issues prior to treatment	PMH: sun exposure and protection	PMH: skincare routine	PMH: aesthetic procedures
1	33	M	Oily	Wrinkles, uneven skin tone, enlarged pores, skin laxity, hyperpigmentation	Barely any sun exposure, moderately excellent sunscreen adherence	Complex skincare routine	Laser procedure unknown date
2	32	F	Oily	Uneven skin tone, enlarged pores, hyperpigmentation	Sun exposure minimal, excellent sunscreen adherence	Moderately complex skincare routine	NAFL procedure half a year prior to study
3	41	M	Mixed	Wrinkles, enlarged pores	Barely any sun exposure, excellent sunscreen adherence	Simple skincare routine	IPL procedure 3 months prior, RF procedure 6 months prior
4	34	F	Mixed	Enlarged pores	Sun exposure moderate, excellent sunscreen adherence	Moderately complex skincare routine	IPL procedure 6 months prior
5	34	M	Oily	Acne scarring, inflammation, enlarged pores	Sun exposure moderate, excellent sunscreen adherence	Moderately complex skincare routine	IPL procedure 6 unknown date
6	43	F	Oily	Wrinkles, uneven skin tone, enlarged pores, skin laxity, hyperpigmentation	Barely any sun exposure, excellent sunscreen adherence	Complex skincare routine	IPL procedure 3 months prior
7	44	F	Sensitive	Wrinkles, uneven skin tone, enlarged pores, skin laxity, hyperpigmentation	Sun exposure minimal, excellent sunscreen adherence	Complex skincare routine	IPL procedure unknown date
8	31	M	Mixed	Wrinkles, uneven skin tone, enlarged pores, skin laxity, hyperpigmentation	Sun exposure minimal, excellent sunscreen adherence	Moderately complex skincare routine	Never had other aesthetic procedures done

*Note:* Sun exposure—barely any: spends daytime indoors, minimal: < 1 h/day, moderate 1–3 h/day; sunscreen adherence—excellent: applies every time before going outdoors; skin care routine—simple: consists of a simple moisturizer, moderately complex: consists of 1–3 steps, complex: consists of over 3 steps. Age refers to age prior to receiving RhCol‐III solution injection.

Abbreviations: F, female; IPL, intensive‐pulse light therapy; M, male; NAFL, non‐ablative fractional laser therapy; PMH, past medical history; RF, radiofrequency therapy.

**FIGURE 1:**
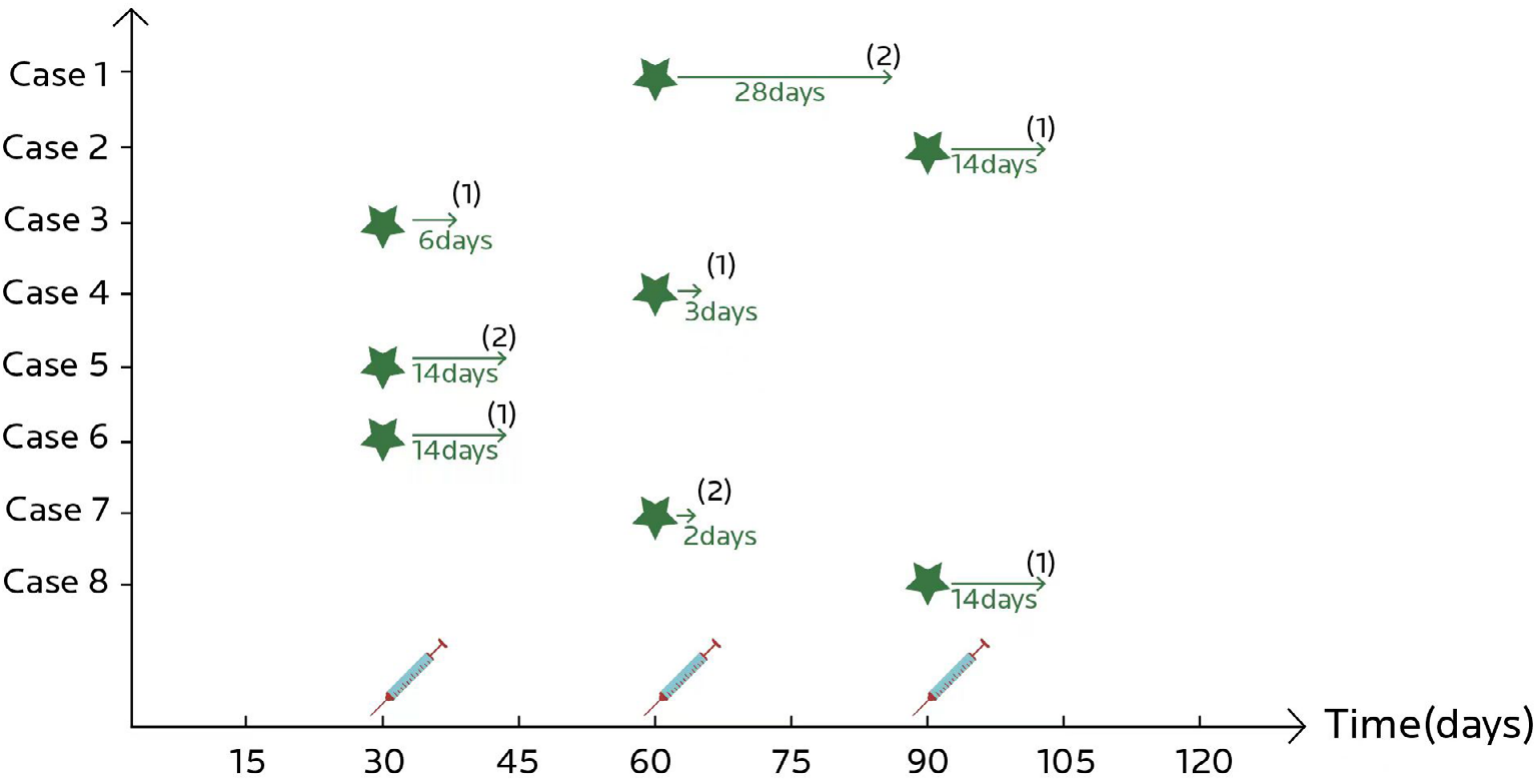
Visual representation of GAIS scores for the eight cases. GAIS, Global Aesthetic Improvement Scale (represented as the numerical value in brackets for each case). Green stars symbolize the specific instance of RhCol‐III solution injection after which the best effect was achieved, whereas the length of the green arrow represents the duration of time that the best effect lasted following injection.

At the end of the study period, five cases reported a “significant improvement” (GAIS score = 1) when judging their overall skin quality brought about by the intervention, whereas three cases stated an “improvement” (GAIS score = 2) with their facial skin (Figure 1).

Apart from short‐term local reactions at the administration site, including mild redness, edema, and tenderness that resolved within 1–3 days postinjection, no significant adverse events were reported during the study period. All participants tolerated the treatment well, with no incidents requiring medical intervention.

### 3.2 Effect of RhCol‐III Solution Injection on Skin Tone and Brightness of Facial Skin

Skin tone evenness measures the uniformity of skin color across the face. Improved evenness reduces visible tone irregularities and discoloration, leading to a more consistent appearance. Skin radiance refers to the skin's brightness and luminosity. Enhanced skin tone evenness often leads to increased radiance. Overall, improvements in this category of skin quality lead to a more vibrant and healthier color.

Improvements in skin tone and brightness were noted as early as 30 days postinjection. An improved skin tone evenness was seen in six cases (6/8, 75%) by the end of follow‐up. On the other hand, the skin radiance index improved in all cases (8/8, 100%) at the end of follow‐up, as compared to baseline (12.9 ± 7.2 vs. 10.8 ± 4.4) (Table 2).

**TABLE 2:** Skin tone evenness, skin radiance, and coloration analysis results of the participants with VISIA imaging after RhCol‐III solution treatment.

Case	Skin tone evenness index				Skin radiance				Individual typology angle (°)			
	Baseline	T1D30	T2D30	T3D30	Baseline	T1D30	T2D30	T3D30	Baseline	T1D30	T2D30	T3D30
1	1.64	1.94 (18%)	1.62 (−1%)	1.52 (−7%)	15.00	9.50 (−37%)	12.50 (−17%)	15.50 (3%)	41.00	ND	ND	41.00 (0%)
2	2.42	1.84 (−24%)	3.1 (28%)	2.17 (−10%)	15.60	25.50 (63%)	24.50 (57%)	26.50 (70%)	59.00	ND	ND	56.00 (−5%)
3	2.79	1.59 (−43%)	1.58 (−43%)	1.36 (−51%)	9.30	9.65 (4%)	5.35 (−42%)	8.96 (−4%)	32.00	ND	ND	38.00 (19%)
4	2.22	2.38 (7%)	2 (−10%)	1.77 (−20%)	16.96	17.13 (1%)	16.03 (−5%)	14.02 (−17%)	29.90	28.90 (−3%)	26.10 (−13%)	29.50 (−1%)
5	4.01	2.89 (−28%)	3.02 (−25%)	2.27 (−43%)	6.98	7.61 (9%)	7.52 (8%)	8.49 (22%)	34.60	36.30 (5%)	36.10 (4%)	39.40 (14%)
6	2.75	2.83 (3%)	3.3 (20%)	3.76 (37%)	10.67	11.55 (8%)	11.73 (10%)	11.85 (11%)	39.40	41.60 (6%)	44.20 (12%)	43.20 (10%)
7	1.82	2.09 (15%)	2.54 (40%)	2.23 (23%)	6.28	6.19 (−1%)	6.25 (0%)	8.29 (32%)	29.90	28.90 (−3%)	26.10 (−13%)	29.50 (−1%)
8	2.2	1.85 (−16%)	1.83 (−17%)	2.11 (−4%)	6.02	6.46 (7%)	6.34 (5%)	5.44 (−10%)	41.90	40.80 (−3%)	42.40 (1%)	41.30 (−1%)
Mean	2.48	2.18 (−12%)	2.37 (−4%)	2.15 (−13%)	10.85	11.70 (8%)	12.79 (18%)	12.92 (19%)	38.40	35.10 (−9%)	34.20 (−11%)	39.40 (3%)
SD	0.74	0.48	0.70	0.73	4.45	6.59	10.10	7.21	9.70	5.20	7.20	8.60

*Note:* Percentage values in brackets refer to the rate of improvement compared to baseline. Skin tone evenness: lower index value indicates an improvement. Skin radiance: higher index value indicates an improvement. Individual typology angle: higher value indicates lighter skin tone.

Abbreviations: RhCol‐III, recombinant type III humanized collagen; SD, standard deviation; T1D30, 30 days post‐first RhCol‐III solution injection; T2D30, 30 days post‐second RhCol‐III solution injection; T3D30, 30 days post‐third RhCol‐III solution injection.

**FIGURE 2:**
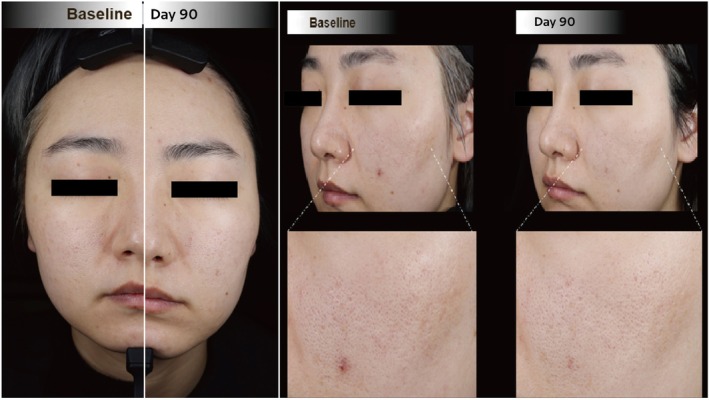
Clinical photographs of a 32‐year‐old female (Case 2) at follow‐up, showing more even coloration and increased skin glow with brighter‐looking skin. A close‐up of the left cheek is provided to emphasize improvement in skin surface evenness through pore number and size reduction.

**FIGURE 3:**
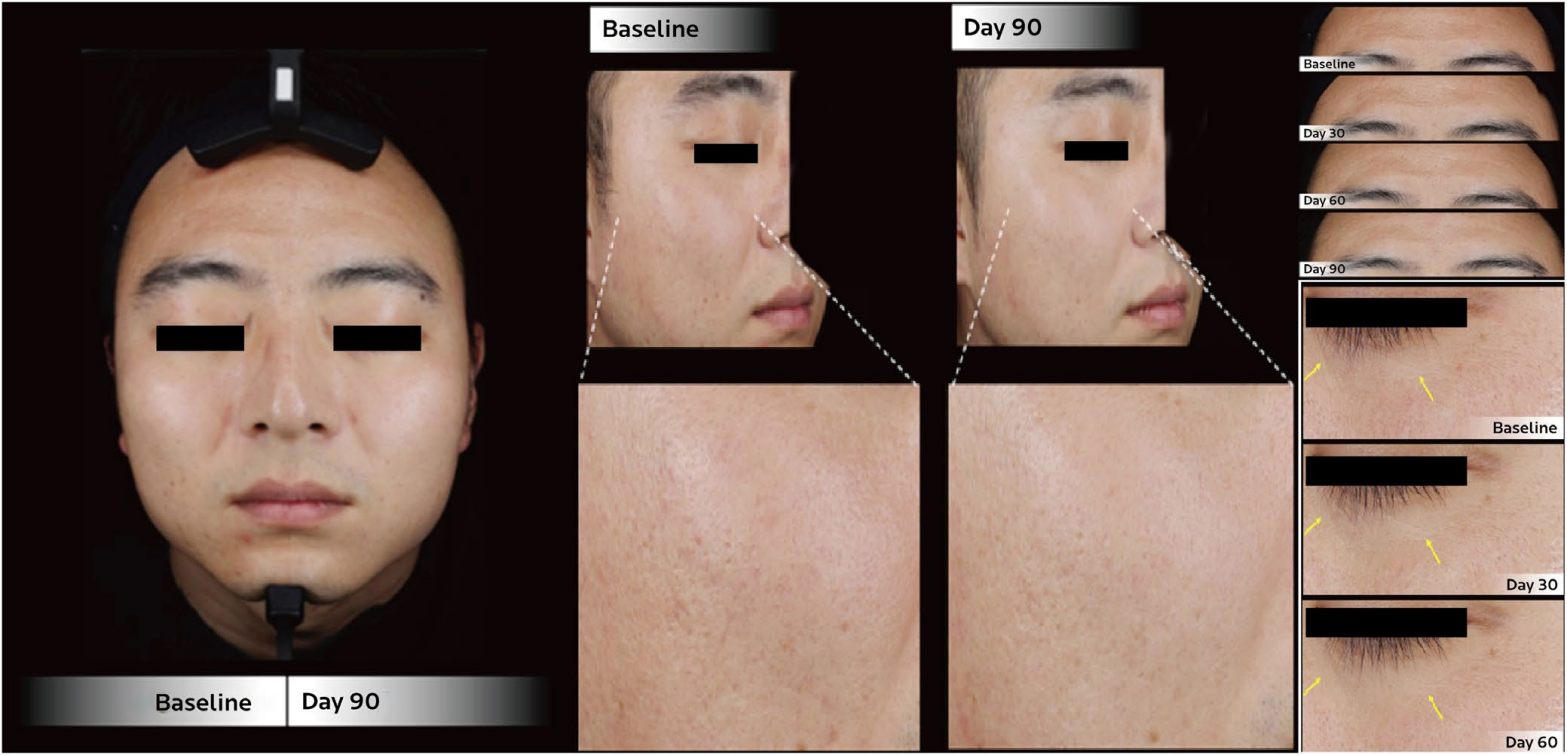
Clinical photographs of a 34‐year‐old male (Case 5) at follow‐up, showing improvement in skin coloration and glow. A close‐up of the right cheek is provided to highlight improvement in skin tone evenness, whereas close‐up of the forehead and periorbital regions were included to demonstrate improved skin surface evenness with fine line reduction in the respective facial areas.

Within this category of skin quality improvements, Cases 2 and 5 stand out, as depicted in Figures 2 and 3, respectively. Case 2 is a representative case in terms of skin radiance improvement, since the index was persistently high at 30, 60, and 90 days post‐baseline (63%, 57%, and 70% improvement, respectively). Case 5 has consistently higher index levels of skin tone evenness, skin radiance, and individual typology angles at all three follow‐up points, when compared to baseline. Both cases had stated that the most prominent skin brightening effect took place within 2 weeks postinjection (Figure 1).

### 3.3 Effect of RhCol‐III Solution Injection on Improving Skin Surface Evenness

Skin surface evenness is yet another key indicator of skin quality, improvements in which often correlate with reduced visible imperfections, leading to a more refined appearance. To be precise, pore size, one of the crucial elements of beauty in the Asian landscape, along with facial wrinkles, being one of the primary signs of aging, were chosen for analysis in the eight cases.

**FIGURE 4:**
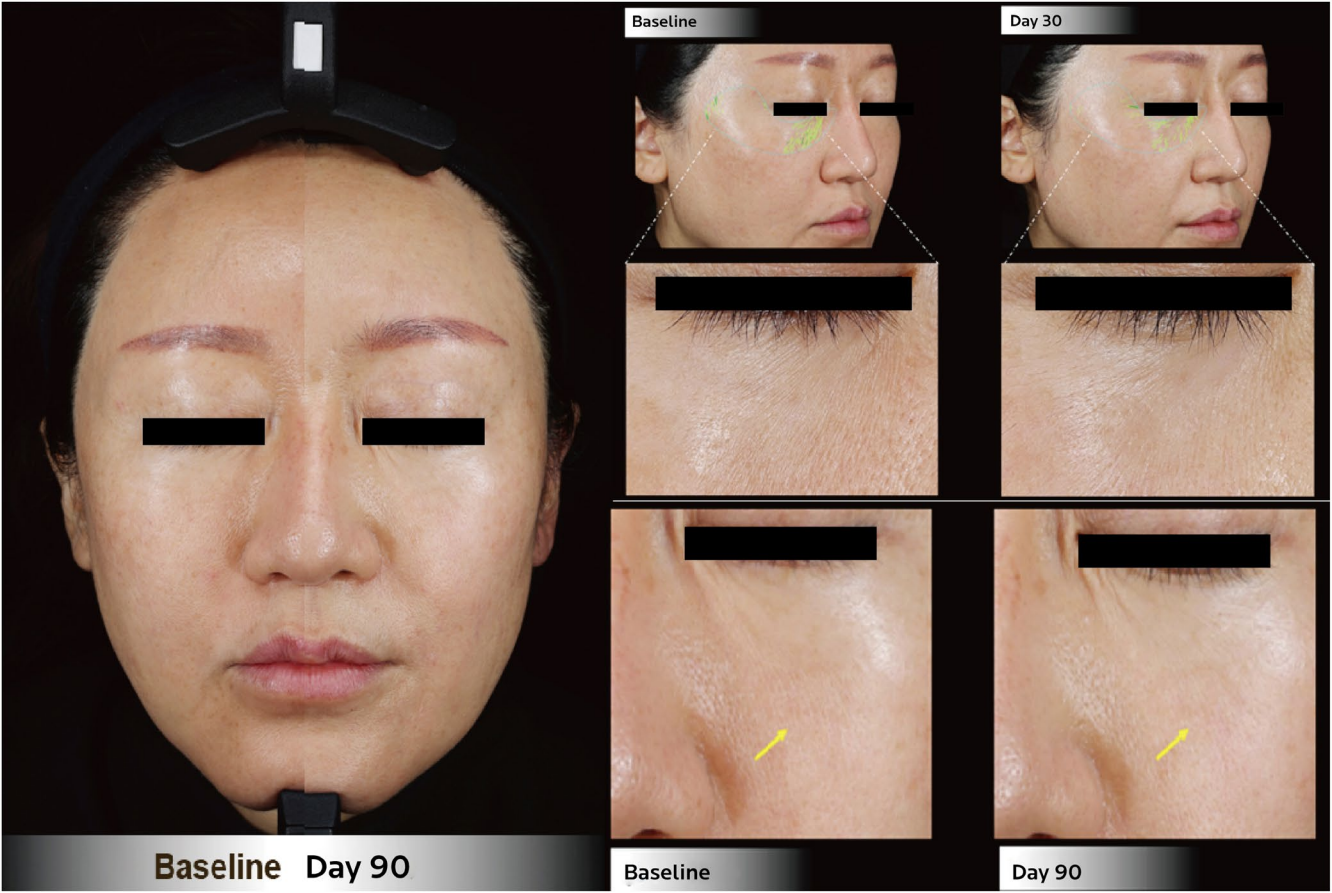
Clinical photographs of a 44‐year‐old female (Case 7) at follow‐up, showing improved skin tone, more even coloration, increased skin glow with brighter‐looking skin. Close‐up images of the periorbital and cheek zones highlight improved skin surface evenness with a reduction in rhytides.

**FIGURE 5:**
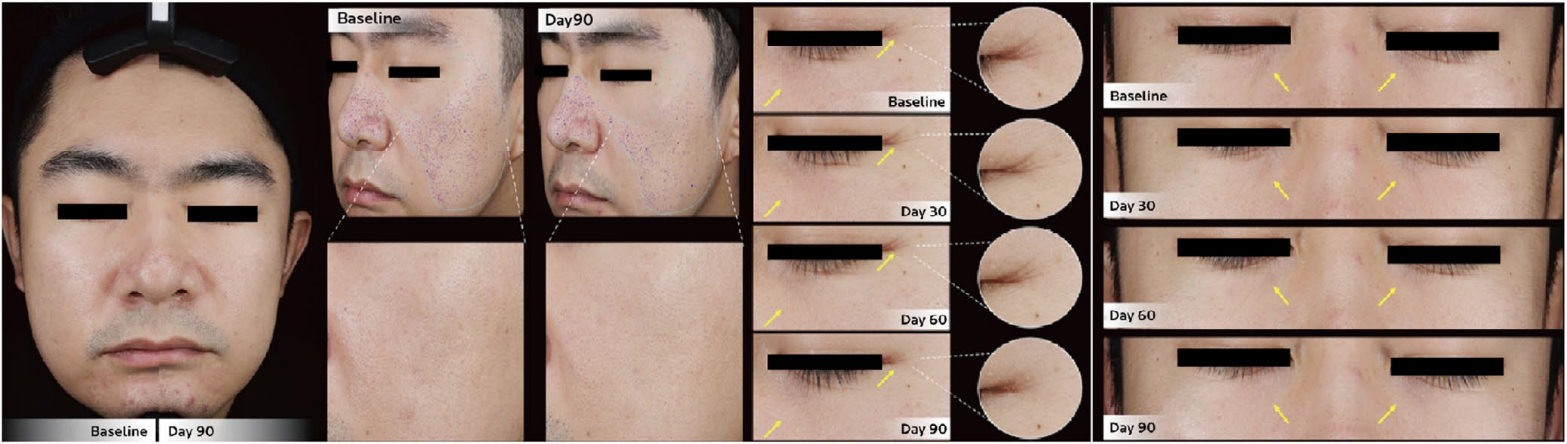
Clinical photographs of a 31‐year‐old male (Case 8) at follow‐up with significant reduction in pore size and number, as shown by the close‐up image of the left cheek. Close‐up images of the left eye showed gradual reduction in crow's feet wrinkles, whereas frontal photographs of the eye region demonstrated continuous improvement in periorbital wrinkles over time.

Pore size and wrinkle reduction were noted in five (5/8, 63%) and six (6/8, 75%) cases, respectively (Table 3). The cases that stood out the most in the aspect of skin surface evenness included Cases 7 and 8 (Figures 4 and 5, respectively). At the end of the follow‐up period, Case 7 demonstrated the largest reduction in this series of cases in wrinkle index (54%) compared to baseline, which can be seen by the improvements in the periorbital and under‐eye wrinkles on VISIA images (Figure 4). The largest reduction in pore size in this case series was achieved in Case 8, decreasing by 20% 90 days post‐baseline, while also having noticeable reductions in crow's feet and under‐eye wrinkles (Figure 5). In addition, Case 5 showed consistent improvement in wrinkle index, while Case 6 also had continuous satisfactory reductions in pore size throughout the follow‐up period. However, variations in individual response were noted when comparing the time taken to achieve the best results in skin evenness improvement (Figure 1). While Cases 5 and 6 reported the best effect to occur within 14 days of the first injection, it occurred within 2 days after the second injection for Case 7 and within 14 days after the third injection for Case 8.

**TABLE 3:** Pores and wrinkles analysis of the participants with VISIA imaging after treatment with RhCol‐III solution injection.

Case	Pore count index				Wrinkle index			
	Baseline	T1D30	T2D30	T3D30	Baseline	T1D30	T2D30	T3D30
1	0.21	0.20 (−5%)	0.20 (−5%)	0.21 (0%)	0.02	0.01 (−22%)	0.02 (−6%)	0.02 (17%)
2	0.23	0.23 (0%)	0.25 (9%)	0.25 (9%)	0.02	0.01 (−47%)	0.02 (−11%)	0.01 (−32%)
3	0.21	0.20 (−5%)	0.18 (−14%)	0.20 (−5%)	0.03	0.02 (−41%)	0.02 (−31%)	0.04 (48%)
4	0.09	0.13 (44%)	0.10 (11%)	0.13 (44%)	0.01	0.01 (27%)	0.00 (−73%)	0.02 (64%)
5	0.14	0.14 (0%)	0.15 (7%)	0.14 (0%)	0.14	0.10 (−25%)	0.10 (−26%)	0.17 (21%)
6	0.16	0.15 (−6%)	0.15 (−6%)	0.15 (−6%)	0.03	0.05 (81%)	0.03 (26%)	0.05 (78%)
7	0.06	0.08 (33%)	0.07 (17%)	0.07 (17%)	0.02	0.04 (63%)	0.02 (−4%)	0.01 (−54%)
8	0.20	0.20 (0%)	0.21 (5%)	0.16 (−20%)	0.03	0.03 (0%)	0.04 (29%)	0.03 (−16%)
Mean	0.16	0.18 (13%)	0.17 (6%)	0.18 (13%)	0.03	0.03 (0%)	0.02 (−33%)	0.03 (0%)
SD	0.05	0.05	0.05	0.05	0.04	0.03	0.01	0.05

*Note:* Percentage values in brackets refer to the rate of improvement compared to the baseline. Pore count: lower index value indicates an improvement. Wrinkle: lower index value indicates an improvement.

Abbreviations: RhCol‐III, recombinant type III humanized collagen; SD, standard deviation; T1D30, 30 days post‐first RhCol‐III solution injection; T2D30, 30 days post‐second RhCol‐III solution injection; T3D30, 30 days post‐third RhCol‐III solution injection.

### 3.4 Effects of RhCol‐III Solution Injection on Dermal Structure and Skin Elasticity

**FIGURE 6:**
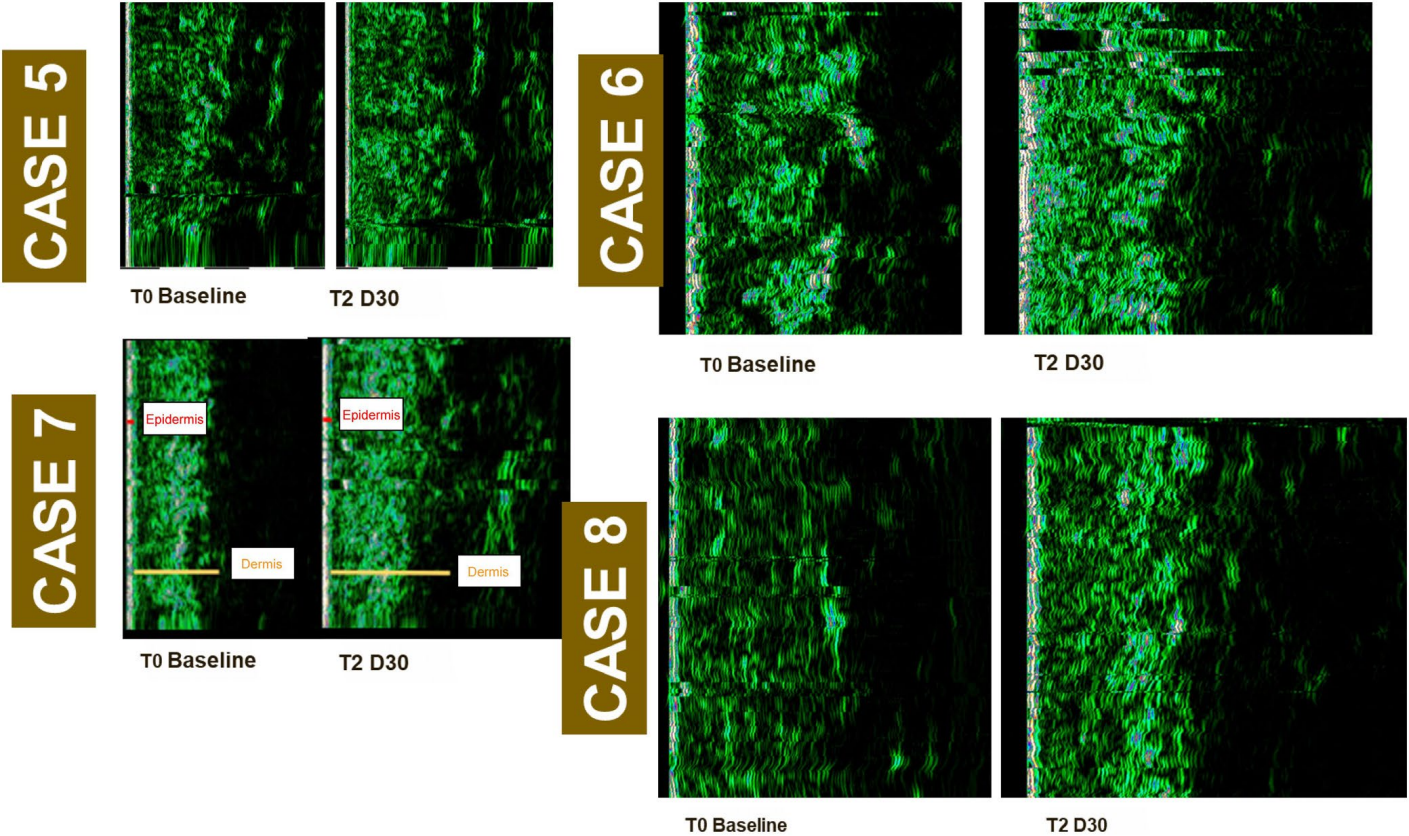
Skin ultrasound images of four cases, taken with UC22, consistent improvements in dermal density and thickness following RhCol‐III injection when comparing baseline and 90 days post‐baseline images. RhCol‐III, recombinant type III humanized collagen.

Owing to varied response times to RhCol‐III solution injection, we aimed to further explore whether deeper structural changes were responsible for the aforementioned discrepancies in superficial skin surface improvements in Cases 5–8. Additional testing with a cutometer and ultrasound was conducted to assess skin elasticity in these cases. At the end of follow‐up, Cases 5–8 showed significant improvements in skin elasticity and firmness, with a mean increase of 33% and 17%, respectively, from baseline (Table 4). Moreover, the mean dermal thickness and density increased by 33% and 18%, respectively, when compared to baseline (Table 5 and Figure 6). Increased dermal thickness often correlates with better elasticity and firmness [19]. The consistent rise in dermal density indicates a positive structural response to treatment, such as improved collagen content, supporting the improvements in skin quality and aesthetic outcomes. Case 8 showed the most substantial improvement in both dermal thickness and density, improving by 97% and 67%, respectively, at the end of the follow‐up period. This increase in thickness is likely linked to the observed enhancements in skin tone, radiance, and overall texture, reinforcing the treatment's efficacy.

**TABLE 4:** Skin elasticity and firmness results of the participants measured with cutometer after RhCol‐III solution treatment.

Case	Elasticity (R2 value)				Firmness (F4 value)			
	Baseline	T1D30	T2D30	T3D30	Baseline	T1D30	T2D30	T3D30
5	0.59	0.58 (−2%)	0.70 (19%)	0.72 (22%)	5.24	5.03 (−4%)	5.10 (−3%)	4.61 (−12%)
6	0.48	0.68 (42%)	0.66 (38%)	0.73 (52%)	4.80	4.28 (−11%)	4.23 (−12%)	4.12 (−14%)
7	0.53	0.65 (23%)	0.65 (23%)	0.70 (32%)	4.78	4.45 (−7%)	4.38 (−8%)	4.03 (−16%)
8	0.59	0.66 (12%)	0.66 (12%)	0.73 (24%)	5.58	4.33 (−22%)	4.89 (−12%)	4.23 (−24%)
Mean	0.54	0.64 (19%)	0.67 (24%)	0.72 (33%)	5.09	4.77 (−6%)	4.65 (−9%)	4.23 (−17%)
SD	0.05	0.03	0.02	0.01	0.32	0.36	0.36	0.29

*Note:* Percentage values in brackets refer to the rate of improvement compared to baseline. Elasticity: higher index value indicates an improvement. Firmness: lower index value indicates an improvement.

Abbreviations: RhCol‐III, recombinant type III humanized collagen; SD, standard deviation; T1D30, 30 days post‐first RhCol‐III solution injection; T2D30, 30 days post‐second RhCol‐III solution injection; T3D30, 30 days post‐third RhCol‐III solution injection.

**TABLE 5:** Dermal thickness and density results of the participants measured with UC22 after RhCol‐III solution treatment.

Case	Dermal thickness (μm)				Dermal density			
	Baseline	T1D30	T2D30	T3D30	Baseline	T1D30	T2D30	T3D30
5	1750.0	1839.0 (5%)	1812.0 (4%)	1930.0 (10%)	11.7	10.4 (−11%)	10.0 (−15%)	13.5 (15%)
6	1430.0	1617.0 (13%)	1638.0 (15%)	1654.0 (16%)	12.0	13.1 (9%)	12.6 (5%)	12.6 (5%)
7	1234.0	1414.0 (15%)	1495.0 (21%)	1703.0 (38%)	15.0	15.9 (6%)	16.0 (7%)	15.7 (5%)
8	883.0	1357.0 (54%)	1698.0 (92%)	1742.0 (97%)	8.4	10.7 (27%)	10.5 (25%)	14.0 (67%)
Mean	1324.3	1556.8 (18%)	1660.8 (25%)	1757.3 (33%)	11.8	12.5 (6%)	12.3 (4%)	14.0 (18%)
SD	314.4	189.5	114.3	104.5	2.3	2.2	2.4	1.1

*Note:* Percentage values in brackets refer to the rate of improvement compared to baseline. Dermal thickness and density: higher index value indicates an improvement.

Abbreviations: RhCol‐III, recombinant type III humanized collagen; SD, standard deviation; T1D30, 30 days post‐first RhCol‐III solution injection; T2D30, 30 days post‐second RhCol‐III solution injection; T3D30, 30 days post‐third RhCol‐III solution injection.

## 4 Discussion

This case series investigated the safety and efficacy of recombinant type III human collagen (RhCol‐III) solution injection for improving skin quality among eight cases with varying photoaging‐related skin concerns, such as wrinkles, uneven skin tone, enlarged pores, skin laxity, and hyperpigmentation. The majority of the cases noticed the positive effect within 1 to 2 weeks after the first injection and achieved the best results by the second injection, indicating a faster time of onset, as compared to topical formulations. This series showed that multiple intradermal injections of RhCol‐III solution were effective in improving key parameters of quality in multiple skin layers: (1) general appearance of the skin surface, which can be judged from a distance through skin tone and brightness; (2) close‐up appearance of the skin surface, assessed by careful inspection of skin smoothness; and (3) underlying deeper tissue structure, evaluated by dermal density and thickness within the skin, which manifests as elasticity and firmness on the surface.

Improvements in elasticity and firmness, taken together with more even skin surface and reduction in wrinkles in most of the cases, suggest that RhCol‐III solution injections may enhance the appearance of the skin by directly increasing collagen content in the dermal layer, promoting collagen dermal remodeling. The progressive increase in dermal thickness and density further supports this hypothesis, indicating the potential of RhCol‐III to promote collagen synthesis or deposition in the skin. These findings are consistent with previous studies that demonstrated that collagen supplementation enhanced skin hydration, elasticity, and dermal density by stimulating fibroblast activity and ECM production [20, 21].

As seen from the satisfaction scores of the cases, the overall skin quality outcomes were positive. However, some variability in treatment response was noted among individual participants, which is expected, owing to the nature of a case series, as a form of scientific communication. This variability could be attributed to individual differences in skin type, skin thickness, hydration levels, baseline skin quality condition, and many other lifestyle factors, such as sun exposure, use of photoprotection, skincare routine, and prior aesthetic procedures, as listed in Table 1. While the majority cases were able to maintain improvements in skin quality parameters throughout the study period, others may have only shown improvements after the first injection or at the end of follow‐up. A potential reason for this may be the natural variability in skin remodeling processes, such as collagen turnover and reorganization, which could fluctuate during the course of treatment.

Since no significant adverse events were reported throughout the study, RhCol‐III solution injections show a favorable safety profile. The absence of allergic reactions or other complications is notable, especially given the potential for immune responses to recombinant proteins. The findings indicate that RhCol‐III is well‐tolerated, making it a promising option for participants seeking nonsurgical skin rejuvenation treatments. In the era of integrated aesthetics, this injection of collagen may be beneficial for combined use with other aesthetic procedures, such as energy‐based devices. As an example, RhCol‐III may be used to increase the integrity of the dermal structures prior to radiofrequency procedures, since the latter may cause much water loss through its thermal effect.

Our findings align with prior research on intradermal collagen injections for skin rejuvenation. A study using type I collagen injections in 20 patients from high‐altitude areas demonstrated significant improvements in skin thickness and a reduction in facial erythema after six treatments. High patient satisfaction and minimal adverse effects were also reported. Compared to this study, our use of RhCol‐III solution for injection showed comparable enhancements in skin texture, firmness, and brightness with only three injections. It is possible that the study by Bin et al. selected cases that had more serious conditions of facial erythema owing to the geographical characteristics of their long‐term place of residence. Our cases were mostly urban residents that presented with common skin conditions that sought aesthetic improvements, rather than needing treatment. Additionally, the differences in onset of action and improvements achieved may be due to the functional differences that type I and type III collagen play in our skin [22]. Another study on cross‐linked hyaluronic acid injections [23] found that such injections fill space and stretch fibroblasts, increasing collagen production thereby grafting the appearance of younger‐looking skin. Injections of RhCol‐III solution could be given together with HA so as to mimic ECM proportions of collagen and hyaluronic acid in the dermis more closely, potentially leading to more efficacious anti‐photoaging results.

The accumulated improvement in skin condition was noted following multiple injections of RhCol‐III, which aligns with the understanding that collagen synthesis is a dynamic process. Initial improvements reported after the first injection may be attributed to the immediate effects of the injected collagen providing structural support and hydration to the skin. Subsequent injections likely stimulate fibroblast activity and enhance endogenous collagen production, leading to more pronounced skin quality improvements over time [24]. This can be explained by the role of RhCol‐III in promoting skin elasticity and structural integrity. The presence of collagen in the ECM is essential for maintaining skin firmness and hydration, which can counteract the effects of UV‐induced damage. Studies have shown that collagen supplementation can improve skin appearance by enhancing dermal thickness and elasticity, thereby reducing the visibility of fine lines and wrinkles [25, 26, 27]. The reduction in inflammation may be linked to the anti‐inflammatory properties of collagen. Collagen can modulate the inflammatory response by promoting the secretion of anti‐inflammatory cytokines and inhibiting pro‐inflammatory mediators [28].

Collagen, particularly human collagen type III, has significant clinical applications in skin rejuvenation therapy due to its essential roles in connective tissue formation and cellular functions. Recent studies have highlighted the potential of RhCol‐III in enhancing wound healing and tissue repair. The high‐resolution crystal structure analysis of a triple‐helix fragment of RhCol‐III has revealed crucial structural features, including charged residues and flexible conformations that facilitate integrin‐mediated cell adhesion. This insight is pivotal for developing recombinant collagen proteins with enhanced membrane adhesion activity. Additionally, RhCol‐III has shown promise in regulating cellular behaviors such as adhesion, proliferation, and migration, addressing the limitations of animal‐derived collagens by minimizing immunogenicity and viral risks [10, 11].

In an in vivo model of UV‐induced skin damage [12], RhCol‐III was applied and compared to saline and un‐crosslinked HA as controls. Over an 8‐week period, RhCol‐III significantly ameliorated photoaging symptoms by reducing epidermal and dermal thickening, enhancing the secretion of collagen type I and III, and promoting ECM remodeling. These findings indicate that RhCol‐III not only contributes to the structural repair of UV‐damaged skin but also supports the refinement and remodeling of critical ECM components.

Recent research underscores the potential of collagen peptides in improving skin color and tone, particularly in the context of photoaging and hyperpigmentation. Collagen peptides have been recognized for their diverse physiological benefits, including moisture retention, antioxidant properties, and inhibition of tyrosinase activity. Tyrosinase, a key enzyme in melanin production, plays a significant role in skin pigmentation disorders. Novel collagen‐derived peptides, such as Asp‐Gly‐Leu (DGL), Gly‐Ala‐Arg (GAR), and Ser‐Asp‐Trp (SDW), have demonstrated potent tyrosinase inhibitory effects, suggesting their efficacy in reducing hyperpigmentation. These peptides interact strongly with the enzyme's active sites, stabilizing their structure through hydrogen bonds and thereby inhibiting melanin synthesis. This mechanism highlights collagen peptides' potential to enhance skin tone and reduce discoloration by targeting the biochemical pathways responsible for pigmentation. Collectively, these findings position collagen peptides as promising agents for improving skin appearance in dermatologic applications [13, 14].

Despite the encouraging findings, the case series has limitations that should be addressed in future research. The small sample size and the lack of a control group limit the generalizability of the results. Further study should involve a controlled, split‐face study that would increase the reliability of the current case series. Further work‐up may include conducting head‐to‐head comparisons of topical versus injectable application modalities of recombinant collagen. Furthermore, the study only included participants of Han ethnicity with Fitzpatrick skin types III/IV, restricting the applicability of the findings to other ethnic groups or skin types. Since the RhCol‐III solution for injection has only been registered in China PR by the National Medical Products Administration (NMPA), we would like to clarify that its use in other countries will need to be preceded by product registration with relevant authorities. Furthermore, due to the follow‐up of cases lasting for several months, there is no way to control their sunscreen usage frequency or the degree of long‐term photodamage that they have suffered. We can only recommend that the participants perform a simple skincare routine, including a mild cleanser, followed by a moisturizer, and finally, a sunscreen, but we cannot be sure that the participant is not using other medications (such as cosmetics or other skin care products) every day. Further areas of exploration can be placed on evaluating the role of the RhCol‐III solution for injection in combination with other injectable or energy‐based aesthetic procedures.

## 5 Conclusion

In conclusion, this study demonstrates that RhCol‐III solution for injection is a safe and effective method for enhancing various aspects of skin quality, including color uniformity, surface smoothness, and elasticity. The absence of adverse reactions further supports the use of RhCol‐III solution as a promising option for nonsurgical skin rejuvenation, especially in Chinese individuals with photoaged skin. Further research with larger and more diverse cohorts is needed to confirm these findings and explore the full potential of RhCol‐III solution in dermatological practice.

## Author Contributions

Y.Y. designed and planned the study; L.T. and Y.Y. conducted data acquisition; L.T. performed data analysis and presentation; L.T. wrote the initial version of the manuscript; L.T. and Y.Y. reviewed the manuscript; Y.Y. prepared the final version of the manuscript.

## Ethics Statement

Informed consent was obtained from all individual participants included in the study.

## Conflicts of Interest

The authors declare no conflicts of interest.

## Data Availability

The data that support the findings of this study are available on request from the corresponding author. The data are not publicly available due to privacy or ethical restrictions.
