# Correction to “Recombinant Type III Humanized Collagen Solution for Injection Improves Skin Photoaging in Chinese Population: A Case Series”

**DOI:** 10.1111/jocd.70396

**Published:** 2025-08-12

Tao L , Yu Y . Recombinant type iii humanized collagen solution for injection improves skin photoaging in chinese population: a case series. J of Cosmetic Dermatology. 2025;24(7):e70276. 10.1111/jocd.70276 40686102 PMC12278026

The author name order for “Yi Yu” was incorrectly displayed as “Yu Yi” in the original publication. The correct name order should read: “Yi Yu”.

We apologize for this error.

